# Prevalence of motorcycle accidents among food delivery drivers and its relation to knowledge, attitudes, and practices in urban areas in Bangkok, Thailand

**DOI:** 10.1371/journal.pone.0303310

**Published:** 2024-05-23

**Authors:** Pusanisa Prakobkarn, Titaporn Luangwilai, Preecha Prempree, Jadsada Kunno

**Affiliations:** 1 Faculty of Medicine Vajira Hospital, Department of Research and Medical Innovation, Navamindradhiraj University, Bangkok, Thailand; 2 Department of Disease Control, Ministry of Public Health, Nonthaburi, Thailand; 3 Office of the Permanent Secretary Ministry of Public Health, Nonthaburi, Thailand; Al Mansour University College-Baghdad-Iraq, IRAQ

## Abstract

Food delivery drivers are at increased risk of motorcycle accidents every year. This study investigated the prevalence of motorcycle accidents among food delivery drivers related to the knowledge, attitudes, and practices in urban areas in Bangkok, Thailand. This was a cross-sectional online survey on motorcycle accidents was distributed among food delivery drivers in urban areas in Bangkok, Thailand from February—March 2023. The study involved 809 participants aged 18 years. A binary logistic regression was conducted to test the association between variable factors and motorcycle accidents, and a Spearman’s analysis was employed to test the correlations between motorcycle accidents and knowledge, attitude, and practice scores. The study found the prevalence of accidents associated with food delivery drivers was 284 (35.1%). The results of the binary logistic regression analysis found that those who drive on an average of more than 16 rounds per day were significantly associated with motorcycle accidents (OR = 2.128, 95%CI 1.503–3.013), and those who had followed improper driving practices were significantly associated with motorcycle accidents (OR = 1.754, 95%CI 1.117–2.752). The correlation analysis found the knowledge score positive significantly with the practice score (r = 0.269, p-value < 0.01) and the attitudes score positive significantly with the practice score (r = 0.436, p-value < 0.01). This study shows the knowledge level correlated with the practice score regarding such accidents. Therefore, our study needs more longitudinal study to identify which variable factors influence motorcycle accidents among FDDs. The current study suggests that the management of traffic safety on urban roads is significantly affected by food delivery services. Thus, this study can be used as baseline data to devise systematic measures to prevent motorcycle crashes of food deivery workers.

## Introduction

Delivery driver jobs are attracting numerous new riders in urban areas worldwide [1,2]. In addition, restaurant businesses are changing their service channels to accommodate online food delivery services, which reflects changing consumption patterns [3]. As an increasing number of consumers now seek to place online food-delivery orders, it is crucial that enough drivers are available to carry out the required deliveries [2]. The increase in the number of transportation rounds may result in fatigue, causing accidents among food delivery drivers (FDDs). Further, another crucial problem has surfaced, where FDDs violate traffic laws by speeding, running red lights, and customizing their cars to satisfy the companies [4].

The recent urban population growth has led to an increase in the number of FDDs, which contributes to an increasing amount of traffic accidents every year. This has a negative effect on the safety of urban areas’ public transit networks [5]. In addition, other factors—such as failure to complete delivery within a specified time, which leads to fines, and large order volumes [5]—cause speeding, leading to accidents. It is also reported that 45% of FDDs receive more than 20 orders per day, and the amount of orders at specific periods has led to a number of traffic safety concerns that are challenging to manage [6]. To work on this, all stakeholders in the food delivery sector, including delivery platforms and drivers, should collaborate to manage the risks in traffic safety associated with food delivery.

In Thailand, food delivery businesses are growing due to comfort-seeking changes in lifestyles. In addition, previous studies have concentrated on increasing road safety for FDDs [7,8]. Previous studies used association guidelines to determine the variables influencing the patterns of accident rates and found that fatal road traffic accidents can be effectively reduced by regulating risky driving behaviors [9]. Risky driving behavior is related to attitudes toward safety and responsibility as well as risk perception [10]. Hence, a better understanding of the knowledge, attitudes, and practices (KAP) on motorcycle accidents is needed among FDDs. Clearly, the increasing prevalence of improper driving practices can have an unfavorable impact on traffic patterns and road safety, which can, in turn, endanger the lives of other motorists and pedestrians [5]. People’s adherence to infection control measures will be largely affected by their KAP. The “KAP theory” is a health behavior change framework wherein factors contributing to human behavioral change are divided into three successive processes, namely the acquisition of proper knowledge, generation of attitudes, and adoption of behaviors (or practices) [11]. The public’s KAP play a major role in the prevention and control of health risks on humans [12]; hence, it is expected that KAP levels will be a deciding factor in the battle against motorcycle accidents among FDDs.

However, studies exploring the relationship between motorcycle accidents among FDDs and KAP are limited in number [13–17]. As the food delivery industry continues to expand globally, it is important to protect FDDs from motorcycle accidents. However, these policies have limited influence, as they are unable to completely manage the risks associated with a driver’s traffic violations [5]. Only a few studies have focused on KAP [13,14,18,19]. In addition, a few focus on the prevalence of motorcycle accidents among FDDs in relation to KAP scores in urban areas in Bangkok, Thailand. On the other hand, the studies done in Malaysia show that 41% of FDDs have been involved in traffic crashes during deliveries [20]. In addition, accidents among delivery drivers increased drastically from 583 in 2014 to 1029 in 2018 [21]. Although delivery motorcycle crash severity was low compared to other motorcycle crashes, the number of patients increased significantly [20–22]. To help address this gap, this paper reports the results of a cross-sectional survey of motorcycle accidents among FDDs in Bangkok, Thailand. Specifically, this study aimed to 1) examine the potential prevalence of motorcycle accidents among FDDs; 2) collect information on FDDs’ baseline KAP related to motorcycle accidents; 3) examine the potential association between variable factors and motorcycle accidents among FDDs; 4) investigate the potential correlation between KAP scores and motorcycle accidents among FDDs. With its findings, this study can help identify various characteristics of FDDs, who are more likely to be exposed to motorcycle accidents. However, a limitation of this study was that the KAP of FDDs with regard to traffic regulations, traffic crashes, and crashes and injuries on delivery motorcycles were not examined; it focused only on accidents instead (the only options were yes or no). Further experimental investigations are needed to estimate these factors as well. The questionnaire responses were based on the perceptions and experiences of a specific group with respect to motorcycle accidents among FDDs.

## Methods

### Study design

This study was conducted using a cross-sectional survey for FDDs in urban areas in Bangkok, Thailand. Data collection took place from 25 February to 31 March 2023. This study was approved by the institutional review board of ethics committee of the Faculty of Medicine Vajira Hospital, Navamindradhiraj University, Bangkok, Thailand. (COA 037/2566).

### Participants

This study was conducted using multistage sampling of FDDs in urban areas in Bangkok following the steps provided. Step I: Stratified sampling by selecting FDDs from 50 districts in Bangkok, which was divided into 6 zones according to the area, economy, society, and lifestyle of the people. Step II: Quota sampling by determining the proportions to find the number of samples of the population in each zone. Step III: Purposive selection by area, covering the economic center and the locations of several places selling food or drinks, such as department stores, shopping centers, food courts, markets, gas stations, or street food restaurants and street food. Through online food ordering applications such as deliver application A (App:A), deliver application B (App:B), deliver application C (App:C), deliver application D (App:D), deliver application E (App:E), and deliver application F (App:F), which receive and deliver food or drinks from a large number of stores. All participants were chosen by convenience sampling and were asked to confirm their voluntary participation. They were also provided instructions for filling out the questionnaire. The included FDDs were over 18 years old. In addition, only healthy people were included in the study.

The sample size was calculated using G*Power (the effect size is 0.116) based on the estimated population of FDDs in urban areas in Bangkok. The Institutional Review Board of the Faculty of Medicine Vajira Hospital is in full compliance with the international guidelines for human research protection according to the Declaration of Helsinki, The Belmont Report, CIOMS Guidelines, and the International Conference on Harmonization in Good Clinical Practice (ICH-GCP). All participants provided written informed consent from before. All participants have been performed in accordance with the Declaration of Helsinki and approved by an appropriate ethics committee.

### Questionnaire

The questionnaire, divided into four sections, takes about 15 minutes to complete. The first part asks for personal information including gender, age, education level, and income per month. The second part asks for the FDDs’ information including motorcycle driving license, experience, type of job, average delivery hours per day, rounds of food delivery per day, speed of driving (km/hr), and how old the motorcycle used to deliver the food is. The third part asks for the type of application the FDDs use.

The question about motorcycle accidents has two options to choose from as the answer: No = 0 (without accidents), Yes = 1 (with accidents including injuries and crashes).

The fourth part asks for KAP level information. The Knowledge section consists of 10 questions (K1–K10), and the responses to the questions were rated on different scales: No = 0 and Yes = 1. The scoring range of the questionnaire was 0 to 10. Knowledge scores for individuals were summed to a total score. FDDs’ overall knowledge was categorized as good if the score was above 80% (≥8 points) and poor if the score was less than 80% (< 8 points). The Attitudes section consisted of 12 questions (A1–A12); the responses to the questions were rated on different scales: Agree = 3, Not Sure = 2, and Disagree = 1. The scoring range of the questionnaire was 0 to 36. The attitude scores for individuals were summed to a total score. The cut-off point by mean was 31. FDDs’ overall attitudes were categorized as favorable if the score was ≥27 points and unfavorable if the score was < 31 points. In addition, the attitudes of FDDs were based on the individual’s knowledge or on the FDD’s experience with motorcycle accidents. The practice section consisted of 17 questions (P1–P17); the responses to the questions were rated on different scales: Practice = 3, Not sure = 2, No practice = 1. The scoring range of the questionnaire was 0 to 51. Practice scores for individuals were summed to a total score. The cut-off point by mean was 46. FDDs’ overall attitudes were categorized as proper practice if the score was ≥18 points and non-proper practice if the score was < 46 points. In addition, the practices of FDDs were based on the individual’s knowledge or on the FDD’s experience with motorcycle accidents. The questions were designed and modified by an expert team of researchers, and the content validity score was 0.70 to 1.00.

### Data collection

Data was collected via an online survey. An invitation was sent asking for the voluntary participation of FDDs and the instructions for filling in the questionnaire.

### Statistical analysis

We summarized the characteristics of the categorical data. Characteristics were compared using descriptive statistics, and categorical data were compared using a chi-square test. Binary logistic regression was used to test the association between variable factors and motorcycle accidents among FDDs. We used Spearman’s correlation between KAP scores among FDDs. A P-value of less than 0.05 was considered to indicate statistical significance. Statistical analysis was performed using the Statistical Package for the Social Sciences Program (SPSS), version 22.

## Results

### Prevalence of accident and characteristics of participants

A total of 809 questionnaire responses were obtained, and FDDs’ variable factors are presented in **Table 1**. The prevalence of accidents among FDDs was 284 (35.1%) or 351 cases in a population of 1,000. The prevalence of accidents was the highest among males, those aged less than 29 years, those with senior high school education, and those with a monthly income of 10,001–20,000 bahts. Other prevalent factors that related FDDs to accidents were the possession of a motorcycle driving license, food delivery experience of more than 3 years, and a main job. Other notable factors included delivering for more than 8 hours per day on average, going for more than 16 rounds per day, driving at a speed of 70 km/hr, and using a motorcycle more than 3 years old for deliveries. App:A and App:F were the most prevalent applications used by FDDs who were related to accidents. The prevalence of knowledge, attitudes, and practice related to accidents was presented as good knowledge, favorable attitudes, and proper practice.

**Table 1:** Prevalence of accident and characteristics of participants among FDDs (n = 809).

Variable factors		FDDs accidents		
		without accidents (n = 525)	with accidents (n = 284)	Prevalence of incidence per 1,000 population
Personal information		n (%)	n (%)	
Gender				
Male		455 (63.8)	258 (36.2)	362
Female		70 (72.9)	26 (27.1)	271
Age (years)				
	≤ 29	213 (62.6)	127 (37.4)	374
	30–39	199 (65.0)	107 (35.0)	350
	≥ 40	113 (69.3)	50 (30.7)	307
Education level				
	Primary/Elementary school	150 (64.4)	83 (35.6)	356
	Junior high school	237 (65.1)	127 (34.9)	349
	Senior high school	65 (60.2)	43 (39.8)	398
	Bachelor’s degree and above	73 (70.2)	31 (29.8)	298
Income per month				
	Below 10,000 bahts	101 (75.4)	33 (24.6)	246
	10,001–20,000 bahts	290 (61.4)	182 (38.6)	386
	Above 20,000 bahts	134 (66.0)	69 (34.0)	340
Food delivery drivers’ information				
Motorcycle driving license				
	No	4 (44.4)	5 (55.6)	349
	Yes	521 (65.1)	279 (34.9)	556
Experience of food delivery drivers				
	≤ 6 months	41 (78.8)	11 (21.2)	212
	> 6 months–1 year	88 (76.5)	27 (23.5)	235
	> 1–3 years	271 (64.8)	147 (35.2)	352
	Above 3 years	125 (55.8)	99 (44.2)	442
Type of job				
	Main job	386 (62.1)	236 (37.9)	379
	Second job	139 (74.3)	48 (25.7)	257
Average food delivery hours per day				
	≤ 8 hours	153 (72.5)	58 (27.5)	275
	> 8 hours	372 (62.2)	226 (37.8)	378
Rounds of food delivery per day				
	≤ 16 rounds	338 (74.3)	117 (25.7)	257
	> 16 rounds	187 (52.8)	167 (47.2)	472
Speed of driving (km/hr)				
	Lower than 70 km/hr	335 (69.5)	147 (30.5)	305
	Above 70 km/hr	190 (58.1)	137 (41.9)	419
How old is the motorcycle used to deliver food?				
	≤ 3 years	429 (65.0)	231 (35.0)	350
	> 3 years	96 (64.4)	53 (35.6)	356
Type of application the FDDs use				
	App:A			
	Without used	508 (65.5)	267 (34.5)	345
	With used	17 (50.0)	17 (50.0)	500
	App:B			
	Without used	227 (62.5)	136 (37.5)	375
	With used	298 (66.8)	148 (33.2)	332
	App:C			
	Without used	311 (67.2)	152 (32.8)	328
	With used	214 (61.8)	132 (38.2)	382
	App:D			
	Without used	461 (67.6)	221 (32.4)	324
	With used	64 (50.4)	63 (49.6)	496
	App:E			
	Without used	486 (65.3)	258 (34.7)	347
	With used	39 (60.0)	26 (40.0)	400
	App:F			
	Without used	503 (66.4)	254 (33.6)	336
	With used	22 (42.3)	30 (57.7)	577
Knowledge, Attitudes, Practice level information				
Knowledge level				
	Poor knowledge	108 (58.1)	78 (41.9)	331
	Good knowledge	417 (66.9)	206 (33.1)	419
Attitude level				
	Unfavorable attitude	82 (61.2)	52 (38.8)	344
	Favorable attitude	443 (65.6)	232 (34.4)	388
Practice level				
	Improper Practices	64 (46.7)	73 (53.3)	314
	Proper Practices	461 (68.6)	211 (31.4)	533

### Knowledge responses on motorcycle accidents among FDDs

Table 2 shows the knowledge of FDDs on motorcycle accidents (items K1–K10). The mean knowledge score was 7.79 ± 1.97, 186 (23.0%) presented poor knowledge and 623 (77.0%) presented good knowledge. The current study demonstrates that most knew that the sign means no overtaking (788 (97.4%))–(K1) and that drinking alcohol is one of the causes of accidents (790 (97.7%))–(K2). In addition, most participants believed that while driving a motorcycle, the turn signal was not required when turning left (722 (89.2%))–(K7), and 679 (83.9%) believed that a flashing yellow light signal meant you should drive faster to pass the area quickly–(K9).

**Table 2:** Frequency scores for knowledge, attitudes, and practice items on motorcycle accidents (n = 809).

Knowledge items on motorcycle accidents		n (%)	
		Yes	No
K1	Know the signs prohibiting overtaking.	788 (97.4)	21 (2.6)
K2	Drinking alcohol is one of the causes of accidents.	790 (97.7)	19 (2.3)
K3	According to Land Traffic Law No. 13 (In Thailand), if the vehicle does not stop, the person crossing the crosswalk will be fined no more than 4,000 baht.	549 (67.9)	260 (32.1)
K4	Motorcyclists in Bangkok go at a speed of 90 km/hr, which is considered legal.	396 (48.9)	413 (51.1)
K5	Motorcyclists can make a U-TURN at the intersection.	263 (32.5)	546 (67.5)
K6	If the driver wants to turn, the vehicle must turn on the turning light at least 30 meters before reaching the turning path.	726 (89.7)	83 (10.3)
K7	Driving a motorcycle when turning left does not require turning on the turn signal.	87 (10.8)	722 (89.2)
K8	Driving with an alcohol level exceeding 50 milligram percent is not considered illegal.	350 (43.3)	459 (56.7)
K9	When seeing a flashing yellow light signal, you should drive faster to pass the area quickly.	130 (16.1)	679 (83.9)
K10	According to Road Traffic Law No. 13 (In Thailand), if driving in reverse, the fine will not exceed 2,000 baht.	632(78.1)	177 (21.9)

Attitude items on motorcycle accidents		n (%)		
		Agree	Not Sure	Disagree
A1	Wearing a helmet correctly every time reduces the severity of the brain injury in the event of an accident.	805(99.5)	0(0.0)	4(0.5)
A2	A helmet that fits snugly on the head does not require a chinstrap when worn.	63(7.8)	37 (4.6)	709(87.6)
A3	Driving in the reverse direction saves time by not having to make a long U-TURN.	122(15.1)	72(8.9)	615(76.0)
A4	Reducing driving speed reduces accidents.	786(97.2)	16(2.0)	7(0.9)
A5	Driving a motorcycle and delivering food while it rains is an opportunity to increase income.	453(56.0)	137(16.9.)	219(27.1)
A6	Driving a motorcycle in Bangkok at a speed of no more than 80 km/hr is safe.	739(91.3)	48(5.9)	22(2.7)
A7	Checking the condition of the car before driving every day wastes time.	133(16.4)	49(6.1)	627(77.5)
A8	Riding a motorcycle when physically exhausted increases the likelihood of an accident.	767(94.8)	27(3.3)	15(1.9)
A9	Drinking alcohol while riding a food delivery motorcycle is normal.	16(2.0)	14(1.7)	779(96.3)
A10	Driving in front of other cars will cause accidents easily.	749(92.6)	20(2.5)	40(4.9)
A11	While in a hurry, you can overtake every lane, which makes it possible to deliver food quickly.	323(39.9)	63(7.8)	423(52.3)
A12	You feel tired and sleepy, but you have to accept the job because the number of food delivery cycles will increase.	346(42.8)	72(8.9)	391(48.3)
Practice items on motorcycle accidents		n (%)		
		Practiced	Not sure	Not practiced
P1	Do you wear a helmet while riding a motorcycle?	793 (98)	14 (1.7)	2 (0.2)
P2	You attach the chinstrap to the helmet and adjust it snugly while riding the motorcycle.	758 (93.7)	44 (5.4)	7 (0.9)
P3	You overtake the car in front by passing on the right side.	549 (67.9)	182 (22.5)	78 (9.6)
P4	You drive in front of other cars in close quarters.	26 (3.2)	219 (27.1)	564 (69.7)
P5	You break through the traffic lights at the intersection when the road is empty	46 (5.7)	307 (37.9)	456 (56.4)
P6	You cross the stop line while waiting for traffic lights.	56 (6.9)	352 (43.5)	389 (49.2)
P7	You strictly follow the traffic rules.	520 (64.3)	241 (29.8)	48 (5.9)
P8	You talk on the phone while riding a motorcycle to deliver food.	120 (14.8)	414 (51.2)	275 (34.0)
P9	You use your phone to view maps and chat with customers while driving.	302 (37.3)	298 (36.8)	209 (25.8)
P10	You have ridden a motorcycle with one hand to deliver food.	52 (6.4)	213 (26.3)	544 (67.2)
P11	You have ridden a motorcycle to deliver food while intoxicated.	13 (1.6)	14 (1.7)	782 (96.7)
P12	You drank alcohol before going out to deliver food.	12 (1.5)	17 (2.1)	780 (96.4)
P13	Speed does not exceed 80 km/hr.	380 (47.0)	286 (35.4)	143 (17.7)
P14	You will overtake other cars in every way that makes it possible to pass.	186 (23.0)	377 (46.6)	246 (30.4)
P15	You check the condition of the car before driving (brakes, wheels, lights).	325 (40.0)	373 (46.1)	111 (13.7)
P16	You ride a motorcycle on the sidewalk.	26 (3.2)	315 (38.9)	468 (57.8)
P17	You increase the number of food delivery rounds per day because the platform has an incentive policy on increasing income or increasing welfare for better medical expenses.	400 (49.4)	180 (22.2)	228 (28.2)

Mean ± SD = 7.79 ± 1.97; Max score = 10, Min score = 0.

Yes = 1, No = 0.

Poor knowledge 186 (23.0%).

Good knowledge 623 (77.0%).

Mean ± SD = 31.08 ± 2.90, Max score = 36, Min score = 22.

Agree = 2; Not Sure = 1; Disagree = 0.

Unfavorable attitudes 134 (16.6%).

Favorable attitudes 675 (83.4%).

Mean ± SD = 46.52 ± 3.05, Max score = 51, Min score = 23.

Practice = 2; Not sure = 1; Not practice = 0.

Improper Practices 137 (16.9%).

Proper Practices 672 (83.1%).

### Attitude responses on motorcycle accidents among FDDs

Table 2 shows the attitudes of FDDs on motorcycle accidents (items A1–A12). The mean knowledge score was 31.08 ± 2.90, 134 (16.6%) presented unfavorable attitudes, and 675 (83.4%) presented favorable attitudes. The study demonstrated that most (805 (99.5%)) agreed that wearing a helmet correctly every time reduces the severity of the brain injury in the event of an accident–(A1), reducing driving speed reduces accidents (786 (97.2%))–(A4), riding a motorcycle when physically exhausted increases the likelihood of an accident (767 (94.8%))–(A8), and driving in front of other cars will cause accidents easily (749(92.6%))–(A10). In addition, most agreed that drinking alcohol while on a delivery motorcycle is not normal (779 (96.3%))–(A9).

### Practice responses on motorcycle accidents among FDDs

Table 2 shows the practice of FDDs in motorcycle accidents (items P1–A17). The mean knowledge score was 46.52 ± 3.05; it presented improper practices (137 (16.9%)) and proper practices (672 (83.1%)). The current study demonstrates that 793 (98%) of them followed proper practices by wearing a helmet while riding a motorcycle–(P1), attaching the chinstrap to the helmet, and adjusting it snugly while riding the motorcycle (758 (93.7%))–(P2). In addition, most did not drink alcohol before going out to deliver food (780 (96.4%))–(P12).

### Association between variable factors and motorcycle accidents among FDDs

According to Table 3, driving more than an average of 16 rounds per day was significantly associated with motorcycle accidents (p-value < 0.001); there was a 2.128-fold increase in motorcycle accidents for such people compared to those driving less than 16 rounds per day (OR = 2.128, 95%CI 1.503–3.013).

**Table 3:** Multivariate analysis of variable factors on motorcycle accidents.

Motorcycle accidents levels				
Variable factors		OR	95%CI	p-value
Gender				0.725
Male		1.097	0.654–1.841	0.725
Female		Ref.		
Motorcycle driving license				0.498
	No	1.654	0.386–7.077	0.498
	Yes	Ref.		
Experience of food delivery drivers				0.260
	≤ 6 months	0.601	0.270–1.341	0.218
	> 6 months–1 year	0.575	0.321–1.031	0.062
	> 1–3 years	0.854	0.582–1.252	0.406
	Above 3 years	Ref.		
Income per month				0.118
	Below 10,000 baht	Ref.		
	10,001–20,000 baht	0.902	0.484–1.680	0.744
	Above 20,000 baht	0.529	0.250–1.033	0.062
Type of job				0.281
	Main job	1.342	0.786–2.292	0.281
	Second job	Ref.		
Average food delivery hours per day				0.535
	≤ 8 hours	Ref.		
	> 8 hours	1.169	0.715–1.912	0.535
Rounds of food delivery per day				< 0.001*
	≤ 16 rounds	Ref.		
	> 16 rounds	2.128	1.503–3.013	< 0.001*
Speed of driving (km/hr)				0.323
	Below 70 km/hr	Ref.		
	Above 70 km/hr	1.188	0.844–1.673	0.323
Knowledge level				0.716
	Poor knowledge	1.078	0.720–1.613	
	Good knowledge	Ref.		
Practice level				0.015*
	Improper Practices	1.754	1.117–2.752	
	Proper Practices	Ref.		

*p -value < 0.05, OR: Odds Ratio, CI: a 95% confidence interval.

0 = without accidents, 1 = with accidents.

Other factors show that practice level was significantly associated with motorcycle accidents (p-value < 0.015). It was seen that there was a 1.754-fold increase in motorcycle accidents among those who followed improper practices compared to those with proper practices (OR = 1.754, 95%CI 1.117–2.752).

### Correlation between knowledge, attitude, and practice scores on motorcycle accidents among FDDs

Table 4 indicates that the knowledge score positive significantly with the practice score (r = 0.269, P-value < 0.01). In addition, the attitudes score positive significantly with practice score (r = 0.436, P-value < 0.01).

**Table 4:** Correlation Spearman’s analysis between knowledge, attitudes, and practices scores.

Correlation coefficient (r)			
Variables	Knowledge	Attitudes	Practice
Knowledge	1.000	-0.067	0.269**
Attitudes	-0.067	1.000	0.436**
Practice	0.269**	0.436**	1.00

**Correlation coefficient significant at 0.01 level (2-tailed).

## Discussion

This study is on the prevalence of motorcycle accidents among FDDs (n = 809) in relation to KAP scores in urban areas in Bangkok, Thailand. Our study presented that the prevalence of motorcycle accidents among FDDs is 35.1% or 351 cases in a population of 1,000. Our study suggested that protection should be provided for self-employed insured persons from employment injuries including occupational diseases and accidents during work-related activities.

The current study showed that the prevalence of motorcycle accidents was the highest among males (362 cases per 1,000 population on motorcycle accidents). Similarly, a previous study showed that 99.2% of the victims were males [20]. In addition, it was shown that these accidents were more prevalent among those younger than 29 years old (374 cases per 1,000 population on motorcycle accidents); a previous study presented that more than half of the injured riders were young riders (less than 29 years old) [21]. In addition, about 65% of riders who experienced being involved in traffic crashes were young riders [22]. Our study also found that many of them were educated until senior high school (398 cases per 1,000 population on motorcycle accidents). From this, it is evident that these riders are inexperienced, lack proper riding skills, and are risk-takers [23]. Although, with an income ranging from 10,001–20,000 bahts/month (386 cases per 1,000 population on motorcycle accidents) among FDDs, our study agrees with previous studies that an increased number of deliveries will increase the income of the riders [21].

The occurrence of accidents depends on other factors as well. Accidents were prevalent among FDDs who worked more than 8 hours a day (378 out of 1,000 cases) and had more than 16 rounds per day (472 out of 1,000 cases). For instance, a study based in Shanghai and Nanjing (China) with 824 delivery riders found that riders have long working days of 9.1 hours on average [1]. In addition, previous studies have shown that long working hours are associated with fatigue, which negatively affects driving and exacerbates the risk of road traffic accidents and injuries [24,25]. Other factors that affected the prevalence were FDDs driving above 70 km/hr (419 per 1,000 cases) and the use of motorcycles older than 3 years (356 out of 1,000). It is also seen that FDDs speed, run red lights, and retrograde their vehicles in an effort to meet the demands of the industry’s “just in time” management process [5,26]. Our study suggested that intervention should be promoted for the regulation of speed and the quality of the motorcycle used. Further, the riding experience was found to be significant, and it was found that elderly motorcyclists, with more riding experience, were less likely to experience a crash compared to younger, less experienced motorcyclists [27]. Hence, the results of our study can be used as baseline data to devise systematic measures to prevent motorcycle crashes of FDDs. Thus, preventative measures should be promoted for novice young part-time workers, on top of safety education/training.

### Association between variable factors and motorcycle accidents among FDDs

In addition, FDDs who drive more than an average of 16 rounds per day were significantly associated with motorcycle accidents (p-value < 0.001). This was shown to be 2.128 times more than those who drive less than 16 rounds per day (OR = 2.128, 95%CI 1.503–3.013). Additionally, too many rounds of food delivery a day might cause fatigue among FDDs. This can affect the driver’s performance by influencing decision-making, causing loss of concentration and control, and delaying reaction time [28]. Furthermore, the time the order is placed basically correlates with the hours of the highest traffic on urban roadways [5]. The increased number of rounds per day might occur from the number of orders, should manage the traffic safety risks of food delivery. In order to address all these concerns, it is essential that all stakeholders in the food delivery marketplace collaborate, including delivery platforms and drivers [29]. Therefore, our study needs more longitudinal research to identify the variable factors that influence motorcycle accidents among FDDs. Moreover, context-specific interventions should be developed, which focus on specific variables affecting the relationship.

Other factors show that practice level was significantly associated with motorcycle accidents (p-value < 0.015). A 1.754-fold increase in motorcycle accidents was seen in those with improper practices (OR = 1.754, 95%CI 1.117–2.752). Previous studies suggest that the prevalence of non-standard helmet use amongst fast food delivery workers was 55.3%. Safety helmets that failed the penetration test had higher odds of being non-standard helmets compared to safety helmets that passed the test [27]. About 74% declared that their employers do not take notice of protective behaviors such as helmet use [30,31]. These results imply that both direct and indirect effects of risky driving behaviors and the number of hours worked can determine the risk of motorcycle accidents [25]. In addition, our study found that attaching the chinstrap to the helmet and adjusting it snugly while riding the motorcycle, which is a good practice, was practiced by 98% of FDDs. In summary, the food-delivery service has a significant impact on urban road traffic safety management. Thus, this study can be used as the baseline data to devise systematic measures to prevent motorcycle crashes of food delivery workers.

### Correlation between knowledge, attitude, and practice scores on motorcycle accidents among FDDs

The study indicates that 419, 388, and 533 per 1,000 cases, respectively, presented with good knowledge, favorable attitudes, and proper practice. Thus, this study, which focuses solely on FDDs from an urban community, showed a correlation between the KAP scores of FDDs with a knowledge score positive significantly with practice score (r = 0.269, P-value = < 0.01). Similarly, others presented that the knowledge regarding the safe use of two-wheeler vehicles was acceptably correlated [13].

In addition, attitudes score moderate positive significantly with practice score (r = 0.436, P-value = < 0.01). Our study agrees with previous studies that risk perception and attitudes towards safety and responsibility are associated with risk [10,19,32]. Furthermore, our study agrees that FDDs’ attitudes influence their intention to obey traffic signals, particularly when they feel that they have a lack of control over work performance [33]. However, in our study, it is found that most FDDs wear a helmet properly while riding a motorcycle, attach the chinstrap to the helmet, and adjust it snugly while riding the motorcycle. Our study agrees with previous studies that the attitude that helmets cannot be protective correlated with not always wearing helmets [34]. In addition, most of them did not practice drinking alcohol before going out to deliver food. Hence, this study can be used as baseline data to devise systematic measures to prevent motorcycle accidents among FDDs. In conclusion, the management of traffic safety on urban roads is significantly affected by food delivery services.

This study highlights the importance of addressing the prevalence of FDD accidents. The data presented 284 (35.1%) or 351 cases in a population of 100. In addition, those who went on more than 16 rounds per day on average and executed improper practices were significantly associated with motorcycle accidents. A special result found that the knowledge level correlated with the practice score regarding such accidents. Therefore, our study needs more longitudinal study to identify which variable factors influence motorcycle accidents among FDDs. Our future goal is to develop health policies for the surveillance of FDD accidents prevention and control in urban communities in Thailand.

## Conclusion

In this study, a survey was conducted on motorcycle accidents among FDDs in urban areas in Bangkok, Thailand. I) It aimed to explore the prevalence of accidents among FDDs was observed to be 35.1%. II) The FDDs who participated in this study evinced overall high KAP scores. III) Our study identified that several influencing factors related to KAP, including driving more than 16 rounds per day on average and following improper practices, were related to motorcycle accidents among FDDs. IV) Thus, this study, which focuses solely on FDDs from an urban community, showed a correlation between the KAP scores of FDDs with a knowledge score positive significantly with practice score. Thus, the food-delivery industry has a significant impact on urban road traffic safety management. Therefore, our study needs more longitudinal research to identify the variable factors that influence motorcycle accidents among FDDs. This study can be used as baseline data to devise systematic measures to prevent motorcycle accidents among FDDs. In particular, we recommend that intervention should be promoted for the regulation of the accidents and quality of the motorcycles FDDs use. Our study suggested that protection should be provided for self-employed insured persons from employment injuries including occupational diseases and accidents during work-related activities.

## Abbreviations

FDDs: food delivery drivers
App:A: Knowledge (K); Attitudes (A); Practices (P); deliver application A
App:B: deliver application B
App:C: deliver application C
App:D: deliver application D
App:E: deliver application E
OR: Odds Ratio
CI: Confidence Interval
SD: standard deviation
